# Retraction: Long noncoding RNA PTPRG-AS1 regulates growth of glioma cells by sponging miR-185-5p

**DOI:** 10.1039/d1ra90057a

**Published:** 2021-02-03

**Authors:** Laura Fisher

**Affiliations:** Royal Society of Chemistry Thomas Graham House, Science Park, Milton Road Cambridge CB4 0WF UK advances-rsc@rsc.org

## Abstract

Retraction of ‘Long noncoding RNA PTPRG-AS1 regulates growth of glioma cells by sponging miR-185-5p’ by Chenyang Xu *et al.*, *RSC Adv.*, 2019, **9**, 10870–10880, DOI: 10.1039/C8RA09546A.

The Royal Society of Chemistry hereby wholly retracts this *RSC Advances* article due to concerns with the reliability of the data. The images in the article, and the raw data provided by the authors, were screened by an image integrity expert. Several western blot control panels contain duplicated bands, including the β-actin bands in Fig. 3B, 3D and 7D. In addition, analysis of the LC3 I band in Fig. 3B shows that the bands may have been inserted onto the background. The raw data provided by the authors was not uncut and unprocessed and therefore could not be used as verifiable raw data to validate the published images.

Furthermore, the raw data provided by the authors was found to closely resemble raw data for a number of other articles, which is unexpected given that there are completely different author lists for these articles.

Given the significance of the concerns about the validity of both the data in the article and the raw data provided by the authors, the findings presented in this paper are not reliable.

Chenyang Xu and Bingqian Ding oppose the retraction. The other authors have been informed but have not responded to any correspondence regarding the retraction.

 

Signed: Laura Fisher, Executive Editor, *RSC Advances*

Date: 19^th^ January 2021

## Supplementary Material

